# Overexpression of *MtDof32* in *Medicago truncatula* enhances leaf and flower organ size through modulation of cell expansion

**DOI:** 10.3389/fpls.2025.1666846

**Published:** 2025-12-04

**Authors:** Tao Guo, Huan Wang, Shumin Wang, Ling Zhou, Shunzhao Sui

**Affiliations:** 1 College of Horticulture and Landscape Architecture, Southwest University, Chongqing, China; 2 Chongqing Engineering Research Center for Floriculture, Key Laboratory of Agricultural Biosafety and Green Production of Upper Yangtze River (Ministry of Education), Southwest University, Chongqing, China; 3 Chongqing Institute of Geology and Mineral Resources, Chongqing, China; 4 Chongqing Key Laboratory of Germplasm Innovation and Utilization of Native Plants, Chongqing Landscape and Gardening Research Institute, Chongqing, China

**Keywords:** *Medicago truncatula*, DOF, transgenesis, organ size, growth and development

## Abstract

As leaf organ size is a key determinant of plant morphology and development, elucidating the cellular and molecular mechanisms governing organ size regulation has become a central research focus in legume biology. *DNA binding with one finger 32* (*MtDof32*), previously identified as a key regulatory factor in organ development, has been shown to influence leaf and flower organ size in *Arabidopsis*. Here, we demonstrated that *MtDof32* overexpression in *Medicago truncatula* resulted in the enlargement of both leaf and flower organs. Cellular analysis revealed that *MtDof32* primarily modulated organ size by controlling cell expansion. Yeast two-hybrid, co-immunoprecipitation (Co-IP), and bimolecular fluorescence complementation (BiFC) assays collectively established the nuclear-localized interaction between MtDof32 and MtEBP1. Additionally, we observed that *MtDof32* overexpression led to delayed flowering and reduced branching in transgenic plants. Comparative analysis with wild-type plants indicated significant alterations in the expression levels of key flowering and branching regulatory genes. This study underscored the conserved role of *MtDof32* in regulating organ size across leguminous species, offering valuable insights for the genetic modification and utilization of this gene in crop improvement strategies.

## 1 Introduction

Legume forage stands out as one of the most economically and nutritionally valuable forage resources, renowned for its strong adaptability, high biomass production, and elevated protein content. It plays a pivotal role in ensuring a stable forage supply (Hamacher et al., 2021). While enhancing forage yield remains a major goal in legume research, a deeper understanding of the genetic and regulatory mechanisms controlling plant architecture, such as leaf size, branching pattern, and resource allocation, is equally critical for targeted crop improvement. Elucidating the molecular pathways that govern organ development and phenotypic plasticity provides a fundamental basis for designing novel strategies to optimize plant form and function, thereby supporting the sustainable development of animal husbandry.

The morphogenesis of plant organs is regulated through multifactorial control systems, among which interspecific genomic divergence constitutes the primary evolutionary determinant governing organ size specification. Consequently, organ size within specific plant varieties tends to be relatively consistent, reflecting the precise regulation of organ growth and development. The ultimate size of plant organs is shaped by the long-term interplay between intrinsic genetic factors and external environmental conditions, involving intricate regulatory pathways (Powell and Lenhard, 2012; Zheng et al., 2023). This complex and finely tuned process engages a multitude of specific genes, including those related to hormones, ubiquitins, cytochromes, microRNAs, transcription factors, and other regulatory elements. To date, numerous growth-promoting and growth-inhibiting factors involved in plant organ development have been identified, providing a foundational understanding of the mechanisms governing organ size regulation. However, while substantial progress has been made in characterizing genes that modulate organ morphogenesis, the underlying regulatory mechanisms remain incompletely understood, and the regulatory network requires further refinement.

Studies at the cellular level have revealed that the plant organs size was governed by the regulation of cell division, differentiation, and growth during organ development, with both cell proliferation and cell expansion playing crucial roles in this process (Cadart et al., 2019). Cell growth serves as a fundamental determinant in the formation, organogenesis, and post-embryonic morphogenesis of plant tissues and organs. Key intermediates in certain cell growth regulatory pathways are essential for intercellular signal transduction, influencing the plant cell cycle, promoting cell differentiation, and indirectly modulating cell size and organ morphology. Furthermore, when cell proliferation is impaired, plants can compensate by increasing cell volume to achieve the mature organ size (Nomoto et al., 2022).

Dof (DNA binding with one finger) family transcription factors, which utilize a single zinc finger motif for DNA binding, are unique to plants and have been identified or predicted in numerous higher plant species (Lohani et al., 2021). These Dof proteins play crucial roles in plant growth and development (Alam et al., 2024; Song et al., 2024). It has been demonstrated that Dof participate in regulating diverse physiological and developmental processes in plants, such as carbon and nitrogen metabolism, flower and seed development, synthesis of secondary metabolites, and vascular and leaf development during plant growth. Notably, one of the key functions of Dof proteins was their involvement in regulating organ development through plant-specific biological processes, often linked to hormone signaling pathways. For instance, overexpression of the tomato gene *SlCDF3* resulted in the enlargement of leaves, petals, and siliques (Corrales et al., 2014). Similarly, overexpression of *OBP3*, a member of the Dof family in *Arabidopsis*, led to delayed development of cotyledons and plants, whereas *obp3* mutants exhibited enlarged cotyledons and plants (Ward et al., 2005). In contrast, plants overexpressing *Dof6* displayed dwarf phenotype, compared to wild-type plants (Rueda-Romero et al., 2012). Interestingly, *MtDof32* exhibited opposite functional characteristics to those of *AtOBP3* and *AtDof6*, as its overexpression in *Arabidopsis* caused enlargement of leaf and flower organs (Guo et al., 2021). Additionally, certain Dof functions were closely associated with hormone signaling pathways. For example, *Arabidopsis AtDof6* regulated abscisic acid (ABA) biosynthesis and metabolism, thereby influencing seed germination. *AtDof6* overexpression disrupted normal plant development, leading to sterility. The high expression levels also conferred delayed progeny seed germination, ABA hypersensitivity, and upregulation of ABA biosynthesis genes. Molecular analyses revealed that AtDof6 physically interacts with TCP14 to form a transcriptional repressor complex that downregulates key genes involved in ABA biosynthesis and metabolism, ultimately inhibiting seed germination (Rueda-Romero et al., 2012). Despite significant progress in understanding the roles of Dof in organ development, the precise mechanisms by which they influence organ size through the regulation of plant cell size remain unclear. Furthermore, whether these processes depend on hormone signaling pathways need to be further investigated.

Our previous studies indicated that, among several predicted Dof transcription factors associated with development, *MtDof32* exhibited the most significant variation in expression levels across various developmental stages of *Medicago truncatula* (*M. truncatula*), which prompted us to select it for further functional analysis. *Arabidopsis* plants overexpressing *MtDof32* exhibited significant enlargement of leaves and flowers (Guo et al., 2021), along with altered expression levels of genes involved in auxin signaling transduction, such as *AtEBP1*, *AtARL*, and *AtKLU*. For functional characterization of *MtDof32* in developmental processes, this study employed transgenic technology to generate *MtDof32*-overexpressing *M. truncatula* plants, building on prior findings. Additionally, using yeast two-hybrid screening, we identified and validated ERBB-3 BINDING PROTEIN 1 (MtEBP1) as an interacting protein of MtDof32. Notably, MtDof32 regulates cell size in *M. truncatula* through its interaction with MtEBP1, a mechanism not previously reported. This study provided a theoretical foundation and genetic resources for elucidating the regulatory mechanisms of organ development in *M. truncatula*, offered new insights for the advancement of high-yield alfalfa biotechnological breeding strategies.

## 2 Materials and methods

### 2.1 Plant materials and growth conditions

In this study, *M. truncatula* (ecotype R108) seeds were employed as the experimental material. The seeds were surface-sterilized with 75% ethanol for 12 minutes, followed by thorough rinsing with sterile distilled water. Subsequently, the sterilized seeds were placed on moistened sterile filter paper and subjected to cold stratification at 4°C for 5 days to break dormancy. After vernalization, the seeds were transferred to standard growth conditions with a photoperiod of 16 h light at 26°C and 8 h dark at 24°C, and maintained until cotyledon expansion. All seeds used in subsequent experiments underwent this standardized pretreatment protocol. Following germination, the seedlings were transferred to Hoagland nutrient solution and cultivated under the same temperature and light conditions (16 h light at 26°C/8 h dark at 24°C) for further growth and development.

### 2.2 Cloning of *MtDof32* from *M. truncatula*

The gene and protein sequences of *MtDof32* (Mtr7g010950) were retrieved from the National Center for Biotechnology Information (NCBI) database and subjected to sequence analysis using MEGA version 6.0 (Koichiro Tamura, Hachioji, Tokyo, Japan). Based on the obtained sequence, a pair of specific primers, Dof32-F/R (see **Supplementary Table 1** for details), was designed to amplify the full-length coding sequence (CDS) from *M. truncatula*. The gene amplification was performed using PrimeSTAR^®^ Max DNA Polymerase (Takara Biotech, Beijing, China) following the manufacturer’s protocol. The resulting PCR products were subsequently cloned into the pEASY-T5 vector (TransGen Biotech, Beijing, China) for further molecular characterization and functional studies.

### 2.3 Quantitative analysis

The R108 *M. truncatula* plants were subjected to phytohormone treatments with 10 μmol/L Indole-3-acetic acid (IAA), 100 μmol/L ABA, 100 μmol/L Gibberellic acid (GA_3_), and 5 mmol/L Salicylic acid (SA) for varying durations. For each phytohormone treatment, samples were collected at 0 h, 2 h, 4 h, 8 h, 12 h, and 24 h, with each treatment replicated three times. The collected samples were immediately flash-frozen in liquid nitrogen to preserve RNA integrity. Total RNA was extracted from the whole plant samples using the Total RNA Kit (Omega, Norcross, USA). Subsequently, cDNA was synthesized from the extracted RNA using the PrimeScript RT Reagent Kit with gDNA Eraser (Takara Biotech, Beijing, China). The first-strand cDNA served as the template for real-time quantitative RT-PCR (qRT-PCR) analysis. The housekeeping gene *Actin* (Mtr3g095530) was used as an internal reference to normalize the expression levels, and the relative expression of *MtDof32* was calculated using the 2^-ΔΔCT^ method. Three biological replicates were performed for each experiment. Primer sequences used in the study were provided in **Supplementary Table 1**. The data are shown as mean values ± SD. Independent t-tests demonstrated that there was significant difference (P<0.05).

### 2.4 Obtaining transgenic plants

The full-length CDS was cloned into the pCAMBIA3302 (p3302) vector, generating the recombinant construct p3302-35S::*MtDof32*. The construct was subsequently introduced into *M. truncatula* calli via Agrobacterium-mediated transformation. Following the selection of PCR-positive transgenic plants, two independent transgenic lines exhibiting high *MtDof32* expression levels were selected for further analysis. To establish stable transgenic lines, T2 generation plants were obtained through self-pollination of primary transformants and used for subsequent functional characterization studies.

### 2.5 Subcellular localization analysis

The *MtEBP1* CDS was cloned into the pSAT-GFP vector using gene-specific primers SAT-MtEBP1-F/R (**Supplementary Table 1**), generating the recombinant construct pSAT-35S::*MtEBP1*-*GFP*. The construct was subsequently introduced into *M. truncatula* leaf protoplasts via polyethylene glycol (PEG4000)-mediated transformation. Subcellular localization of the MtEBP1-GFP fusion protein was analyzed using laser scanning confocal microscopy 16–24 h post-transformation. Protoplast isolation and transformation were performed according to established protocols (Yoo et al., 2007).

### 2.6 Yeast two-hybrid assay

The *MtDof32* CDS was amplified by PCR using pGBKT7-Dof32-F/R primers and subsequently fused into the pGBKT7 vector through the action of endonucleases and seamless ligases, generating the bait construct pGBKT7-Dof32. The recombinant plasmid was transformed into the Y187 yeast strain for library screening. Yeast two-hybrid screening was conducted using the Matchmaker GAL4 Two-Hybrid System (Clontech, USA), where the Y187 strain carrying the bait construct was mated with AH109 strain containing a *M. truncatula* cDNA library for protein interaction screening.

For prey vector construction, the *MtEBP1* CDS was cloned into pGADT7 and transformed into yeast strain AH109 according to the manufacturer’s instructions (Clontech, USA). Protein-protein interactions were assessed by co-culturing the transformed Y187 and AH109 strains on selective media (SD/-Ade/-Trp and SD/-Ade/-His/-Leu/-Trp). Following incubation at 30°C for 48 h, positive colonies were subjected to β-galactosidase activity assays to confirm protein interactions.

### 2.7 Bimolecular fluorescence complementation assay

*MtDof32* CDS was fused to YFP at its N-terminus in the pSYNE vector, generating the 35S promoter driven pSYNE-35S::*MtDof32* construct. Similarly, the *MtEBP1* CDS was inserted into the pSYCE vector with a C-terminal YFP tag, creating the pSYCE-35S::*MtEBP1* fusion construct. Both recombinant plasmids were introduced into *A. tumefaciens* strain GV3101 and subsequently infiltrated into the abaxial epidermis of *Nicotiana benthamiana* leaves using agroinfiltration. YFP fluorescence was visualized 48 h post-infiltration using laser scanning confocal microscopy.

### 2.8 Co-immunoprecipitation assay

Tobacco leaves from one-month-old plants were infiltrated with *Agrobacterium tumefaciens* carrying 35S::*MtDof32*-FLAG and 35S::*MtEBP1*-GFP constructs. Two days post-infiltration, leaf samples were harvested, immediately frozen in liquid nitrogen, and stored at -80°C. The frozen tissue was ground to a fine powder in liquid nitrogen and homogenized in IP lysis buffer supplemented with a protease inhibitor cocktail. The homogenate was centrifuged at 15,000 × g for 10 min at 4°C, and the resulting supernatant was collected as the total protein extract. The total protein extract was incubated with pre-treated beads at 4°C to facilitate binding of the target protein. After incubation, the beads were washed with ice-cold PBS to remove non-specifically bound proteins. Subsequently, the beads were collected and resuspended in 45 μL of IP lysis buffer and 15 μL of 4× SDS loading buffer. The mixture was boiled at 100°C for 5 minutes to elute the proteins, followed by centrifugation at 15,000 × g for 5 minutes. The resulting supernatant, designated as the IP sample, was collected and stored at -80°C for subsequent analysis. An input sample was prepared by mixing 45 μL of the total protein extract with 15 μL of 4× SDS loading buffer, followed by denaturation at 100°C for 5 min. The sample was then stored at -80°C. For immunoblotting analysis, the input and IP samples were probed with anti-GFP and anti-FLAG antibodies to detect the respective proteins.

## 3 Result

### 3.1 Identification and homology analysis of MtDof32 in *M. truncatula*

**Figure 1:**
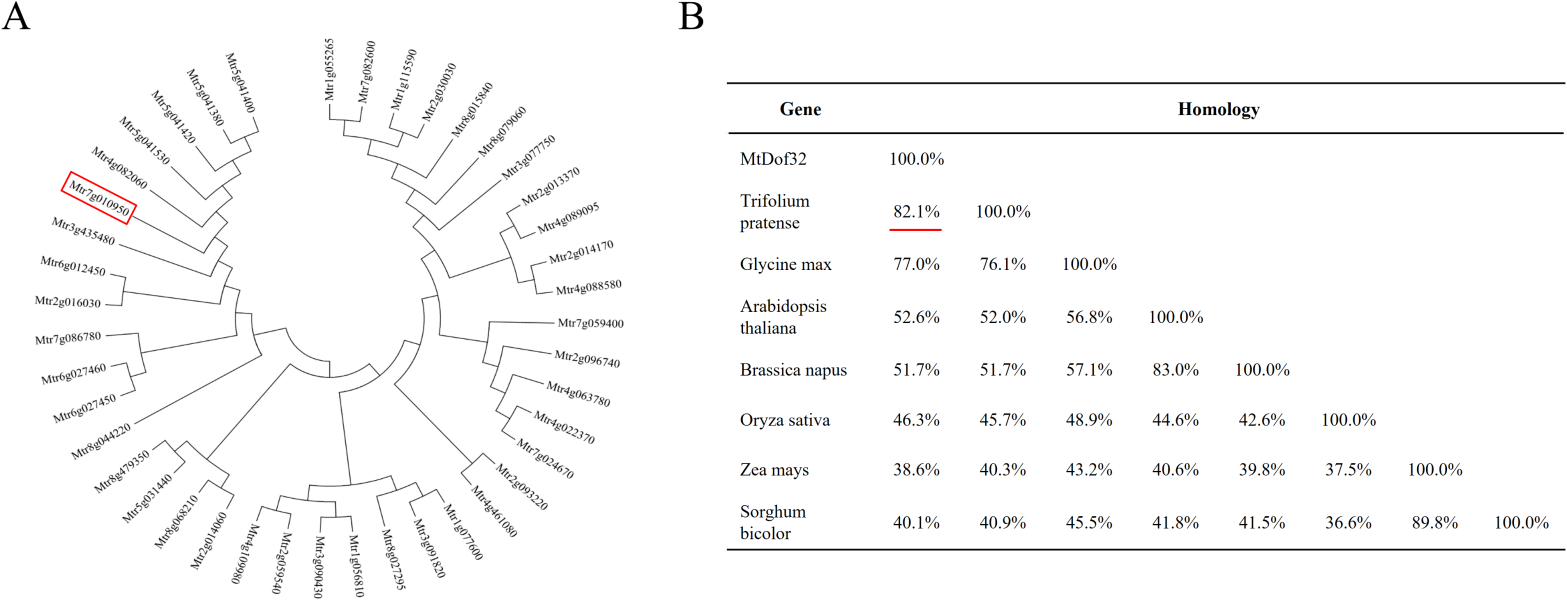
Dof transcription factors phylogenetic tree analysis. **(A)** Phylogenetic tree analysis between Dof proteins from *M. truncatula*. Dof32 is circled by a red box. **(B)** Sequence homology analysis of Dof proteins across selected species was conducted based on protein sequences acquired from the National Center for Biotechnology Information (NCBI). Gene accession numbers: *M. truncatula* Dof32 (Mtr7g010950), *Trifolium pretense* (L195_g008848), *Glycine max* (Gm18G260500), *Arabidopsis thaliana* (At5g39660), Brassica napus (Bn04g09490D), *Oryza sativa* (Os01g15900), *Zea mays* (Zm2g162749), *Sorghum bicolor* (Sb1g045840).

Structural analysis revealed that MtDof32 protein (Mtr7g010950) possessed a single characteristic Dof domain, confirming its classification within the Dof transcription factor family. We systematically classified 42 Dof family members in *M. truncatula* based on conserved motif organization and constructed a phylogenetic tree using full-length protein sequences (**Figure 1A**). Sequence homology analysis between MtDof32 and Dof proteins from other species was performed using MEGA6.0 software (**Figure 1B**). Comparative analysis demonstrated that MtDof32 shared the highest sequence identity (82.1%) with L195_g008848, a Dof protein identified in *Trifolium pratense*. Based on these findings, we hypothesize that both functional redundancy and divergence may exist between MtDof32 and other transcription factors in the Dof family.

### 3.2 Expression analysis of *MtDof32* in *M. truncatula*

**Figure 2:**
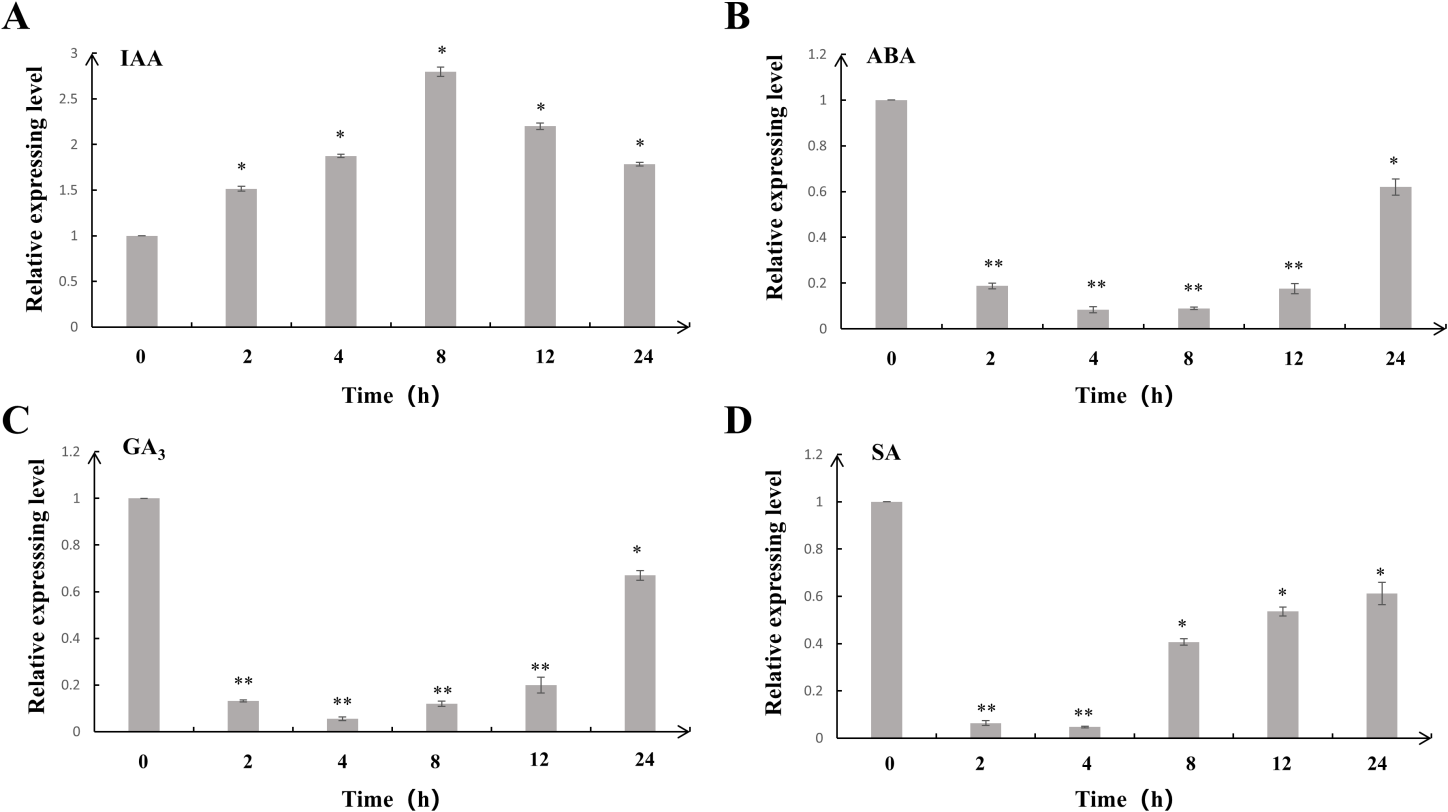
Analysis of expression patterns of *MtDof32* under exogenous hormones treatment. Three-week-old *M. truncatula* seedlings were treated with **(A)** 10 μmol/L IAA, **(B)** 100 μmol/L ABA, **(C)** 100 μmol/L GA_3_, and **(D)** 5 mmol/L SA via foliar application. Samples were collected at 0, 2, 4, 8, 12, and 24 h post-treatment. Data represent mean ± SD of three biological replicates. *denotes statistically significant differences at p < 0.05. **denotes statistically significant differences at p < 0.01.

The regulation of plant growth, developmental processes, and stress responses is mainly mediated by plant hormones. To further investigate the response of *MtDof32* to exogenous hormones, we analyzed its expression patterns under various treatments (**Figure 2**). Quantitative analysis demonstrated that under exogenous IAA treatment, the expression level of *MtDof32* initially increased, peaking at 8 h, before subsequently declining. In contrast, treatments with exogenous ABA, GA3, and SA led to significant downregulation of *MtDof32* expression within 24 h. These findings suggested that the transcriptional activity of *MtDof32* was closely associated with IAA, ABA, GA3, and SA, implying its possible regulatory function in various hormone-mediated signaling cascades.

### 3.3 Ectopic expression of *MtDof32* led to enhanced organ size in *M. truncatula*

**Figure 3:**
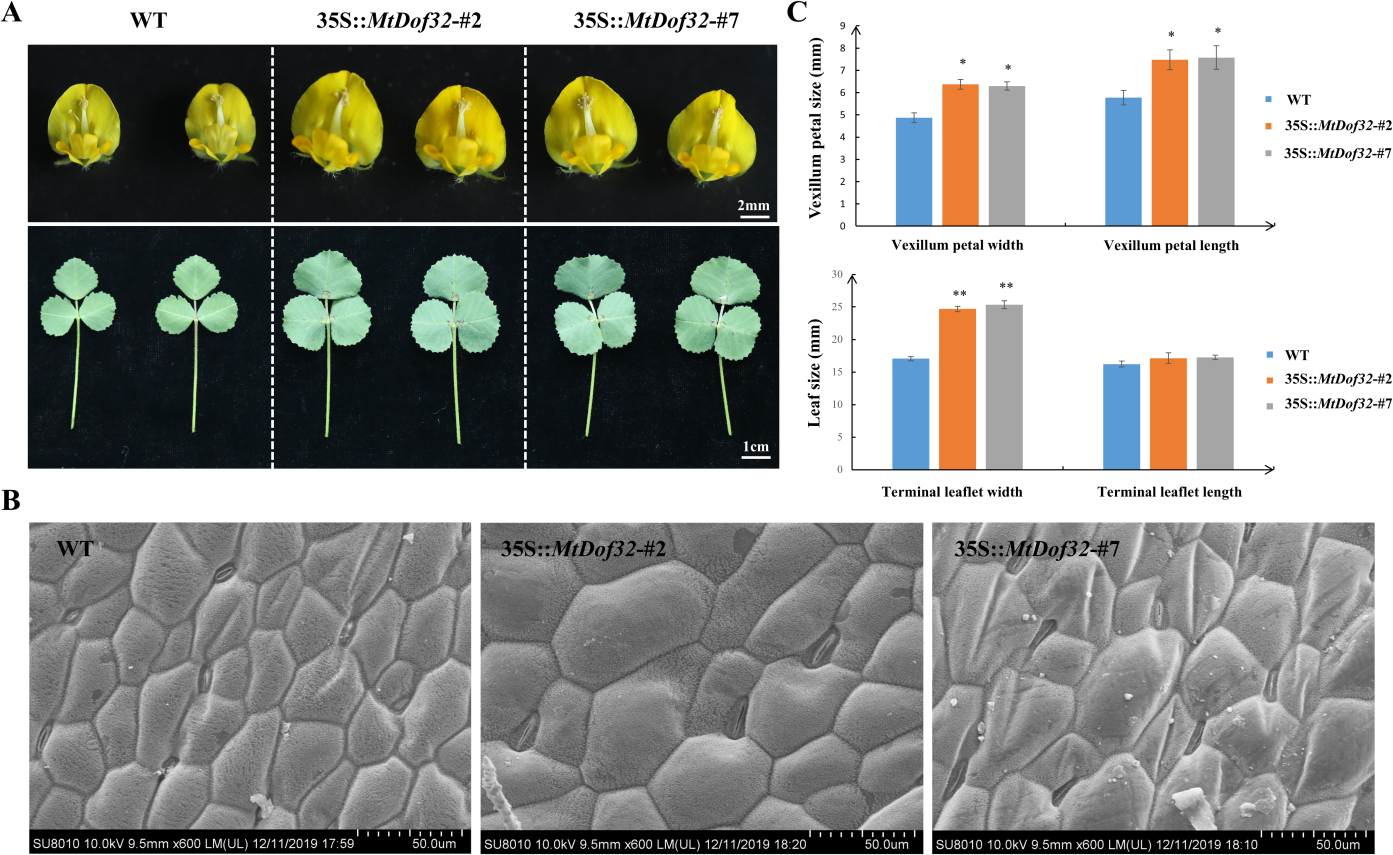
Comparative analysis of leaf and flower between *MtDof32* transgenic plants and wild *M. truncatula.***(A)** Comparative analysis of leaf and flower between *MtDof32* transgenic plants and wild *M. truncatula.* Samples were collected at 60 days of growth. **(B)** Differences of the epidermal mesophyl cells between the wild-type and *MtDof32* overexpressed transgenic *M. truncatula* in scanning electron microscopy. **(C)** Comparison of terminal leaflet and vexillum petal size between wild type and *MtDof32* transgenic group *M. truncatula* (n=18). *denotes statistically significant differences at p < 0.05. **denotes statistically significant differences at p < 0.01.

Ectopic expression of *MtDof32* in *M. truncatula* promoted the enlargement of both flower and leaf organs (**Figure 3**). At 60 days after planting, the average width of the vexillum petal in the transgenic lines MtDof32-#2 and MtDof32-#7 was 6.29 mm and 6.37 mm, respectively, compared to 4.87 mm in wild-type plants. Additionally, the average length of the vexillum petal was 7.45 mm and 7.57 mm, respectively, significantly exceeding the 5.75 mm of the wild type. Similarly, in these two transgenic lines, the average width of the apical leaflets in trifoliate compound leaves was 2.47 cm and 2.53 cm, respectively, significantly larger than the 1.71 cm measured in the wild type. A similar trend in the enlargement of both leaf and floral organs was observed as early as 30 days after planting (**Supplementary Figure 3**). To further investigate the cellular basis of these phenotypic changes, scanning electron microscopy was employed to examine the size of leaf cells in *MtDof32*-overexpressing *M. truncatula*. Microscopic analysis demonstrated that transgenic plants exhibited substantially larger leaf cells than those of wild-type R108, suggesting that *MtDof32* likely influences leaf size by promoting cell enlargement.

### 3.4 Overexpression of *MtDof32* delayed the flowering time of *M. truncatula*

**Figure 4:**
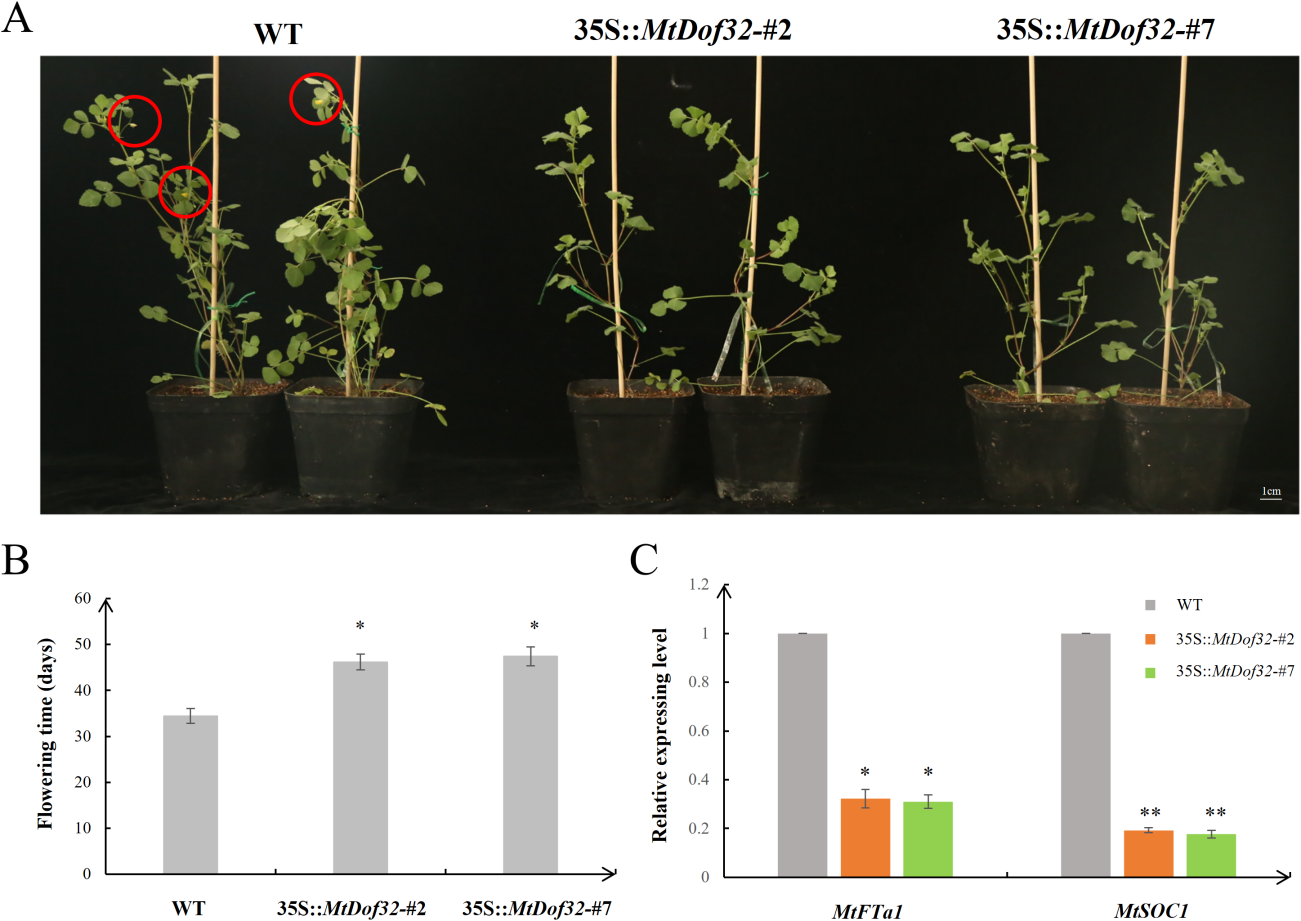
Phenotypic comparison of flowering time in *MtDof32*-overexpressing transgenic lines versus wild-type *M. truncatula.***(A)** Phenotypic comparison analysis of *MtDof32* transgenic lines and WT at 45 days of growth **(B)** Flowering time phenotype comparison in *MtDof32* transgenic lines versus WT (n=10). *denotes statistically significant differences at p < 0.05. **(C)** Quantitative expression analysis of *MtFTa1* and *MtSOC1* genes in *MtDof32* transgenic lines and WT using qRT-PCR. Data represent mean values ± SD from three biological replicates. *denotes statistically significant differences at p < 0.05. **denotes statistically significant differences at p < 0.01.

*MtDof32* transgenic *M. truncatula* exhibited a delayed flowering phenotype, with flowering time occurring 8–11 days later than wild-type controls (**Figure 4**). *MtFTa1* (Flowering Locus T) and *MtSOC1* (Suppressor of Overexpression of Constans 1) serve as pivotal integrating factors in regulating flowering time. Consequently, we examined the expression levels of these two crucial genes in both transgenic and wild-type plants. Analysis of gene expression revealed significant downregulation of *MtFTa1* and *MtSOC1* transcripts in *MtDof32*-overexpressing plants. These findings suggested that *MtDof32* functioned as a key regulator of flowering time in *M. truncatula*.

### 3.5 Ectopic expression of *MtDof32* reduced the number of primary branches in *M. truncatula*

**Figure 5:**
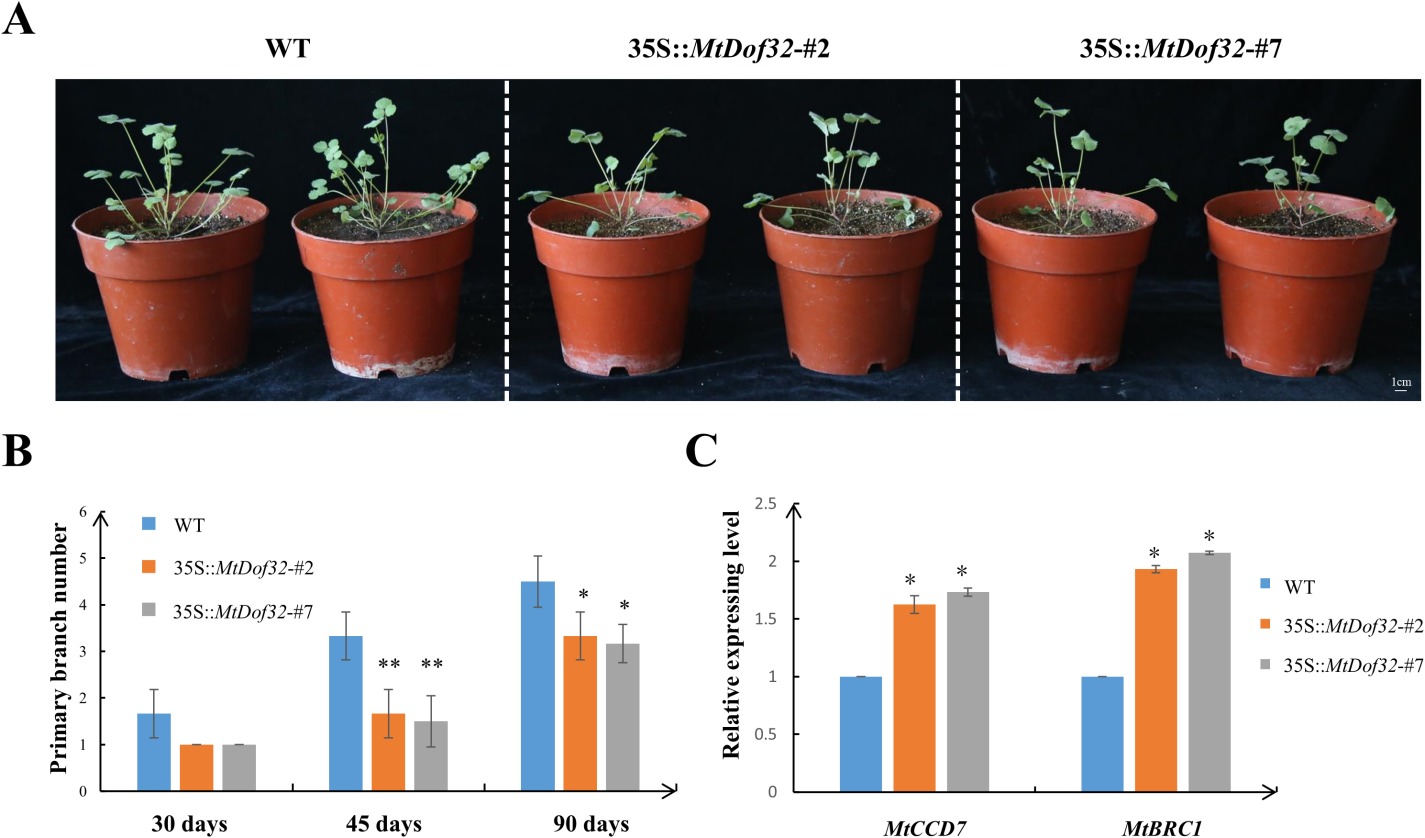
Comparative analysis of primary branches number in *MtDof32* transgenic lines and wild *M. truncatula.***(A)** Comparative analysis of *MtDof32* transgenic plants and wild *M. truncatula* at 30 days of growth. **(B)** Differences of the primary branches number between the wild-type R108 and *MtDof32* overexpressed transgenic *M. truncatula* (n=18). *denotes statistically significant differences at p < 0.05. **denotes statistically significant differences at p < 0.01. **(C)** Quantitative expression profiling of branching-related genes in transgenic and WT plants using qRT-PCR. Data are presented as mean ± SD from three independent biological replicates. *denotes statistically significant differences at p < 0.05.

After the cotyledons unfolded, the seedlings were transferred to soil, and the time of plant growth was initiated. The number of branches in *MtDof32*-overexpressing *M. truncatula* was recorded at 30, 45, and 90 days of growth (**Figure 5**). At 30 days, overexpressing *MtDof32* transgenic *M. truncatula* exhibited an average of 0.7 fewer primary branches compared to wild-type *M. truncatula*. At 45 days, the WT had an average of 1.7-1.8 more primary branches than the *MtDof32*-overexpressing transgenic lines. At 90 days, the average difference in branch number was 1.2-1.4. These results demonstrated that transgenic *M. truncatula* plants exhibited a significant reduction in primary branch number compared to R108 wild-type plants, a phenotype consistent with that observed in transgenic *Arabidopsis*. qRT-PCR analysis showed that the relative expression levels of both *MtCCD7* and *MtBRC1* genes in *MtDof32*-overexpressing transgenic *M. truncatula* lines (#2 and #7) were significantly upregulated compared to the WT control. It is noteworthy that although *MtDof32*-overexpressing transgenic plants exhibited a significant increase in leaf size, their total aerial biomass was significantly lower than that of wild-type controls at maturity (**Supplementary Figure 4**). This reduction in biomass is attributable to the decreased branch number in the overexpression lines, highlighting a notable trade-off between leaf cell expansion and branching capacity under constitutive *MtDof32* expression.

### 3.6 MtDof32 were involved in interactions with MtEBP1

Using pGBKT7-MtDof32 as the bait protein, we performed a yeast two-hybrid screen against a cDNA library derived from *Medicago truncatula*. After multiple rounds of stringent selection on quadruple-dropout medium (SD/–Trp/–Leu/–His/–Ade) supplemented with X-α-Gal, a number of candidates interacting proteins were identified. All candidate proteins identified in the yeast two-hybrid screen are listed in **Supplementary Table 2**. Among the candidate genes obtained from the screen, many encoded proteins of unknown function or were not directly relevant to the focus of this study. Therefore, we focused further validation and analysis on MtEBP1 (Mtr7g069390), a candidate with well-established biological roles and clear functional relevance to our investigation.

**Figure 6:**
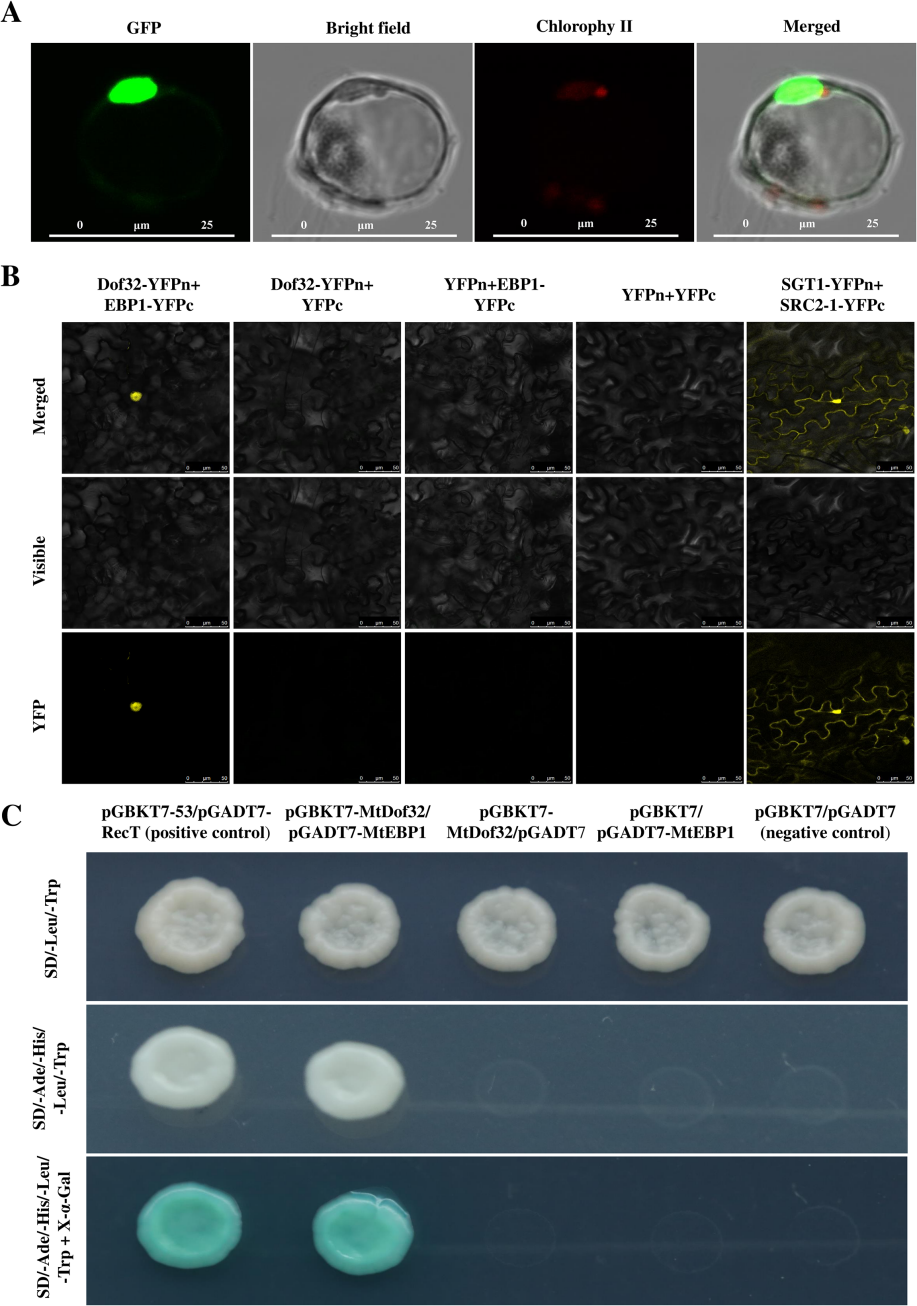
Subcellular localization of the MtEBP1, and proteins interaction between MtDof32 and MtEBP1. **(A)** Subcellular localization of MtEBP1 in the protoplast cell of *M. truncatula*. Scale bars = 25 µm. **(B)** The protein-protein interaction between MtDof32 and MtEBP1 was examined using BiFC analysis in tobacco epidermal cells. SGT1-YFPn+SRC2-1-YFPc was used as a positive control for the BiFC system. Scale bars = 50 µm. **(C)** Yeast two-hybrid assays were conducted to examine the direct interaction between MtDof32 and MtEBP1 proteins.

To investigate the subcellular localization of MtEBP1, 35S::*MtEBP1*-GFP construct was generated and transiently expressed in *M. truncatula* protoplasts to determine MtEBP1 localization. Confocal microscopy revealed nuclear localization of the fusion protein (**Figure 6A**), confirming MtEBP1 as a nuclear protein, consistent with bioinformatics predictions. Since the nuclear localization of MtDof32 has been previously demonstrated (Guo et al., 2021), we will not elaborate on it here.

The physical interaction between MtDof32 and MtEBP1 was subsequently confirmed through BiFC assays. Co-expression of pSYNE-MtDof32 and pSYCE-MtEBP1 in tobacco epidermal cells resulted in distinct yellow fluorescence signals specifically localized to the nucleus (**Figure 6B**), confirming their interaction within the nucleus.

The protein-protein interaction between MtDof32 and MtEBP1 was further validated using yeast two-hybrid assays. The yeast strains Y187 (containing pGBKT7-MtDof32) and AH109 (containing pGADT7-MtEBP1) exhibited normal growth on SD/-Ade -His-Leu-Trp selection medium, comparable to the positive control (pGBKT7-53 + pGADT7-RecT). Furthermore, these colonies demonstrated β-galactosidase activity, as evidenced by blue coloration on SD/-His-Leu-Trp-Ade medium supplemented with X-α-Gal (**Figure 6C**).

The CoIP assays, performed by co-expressing 35S::*MtDof32*-FLAG and 35S::*MtEBP1*-GFP in tobacco leaves, confirmed that FLAG-tagged MtDof32 specifically co-precipitates with GFP-tagged MtEBP1 *in vivo* (**Supplementary Figure 5**).

**Figure 7:**
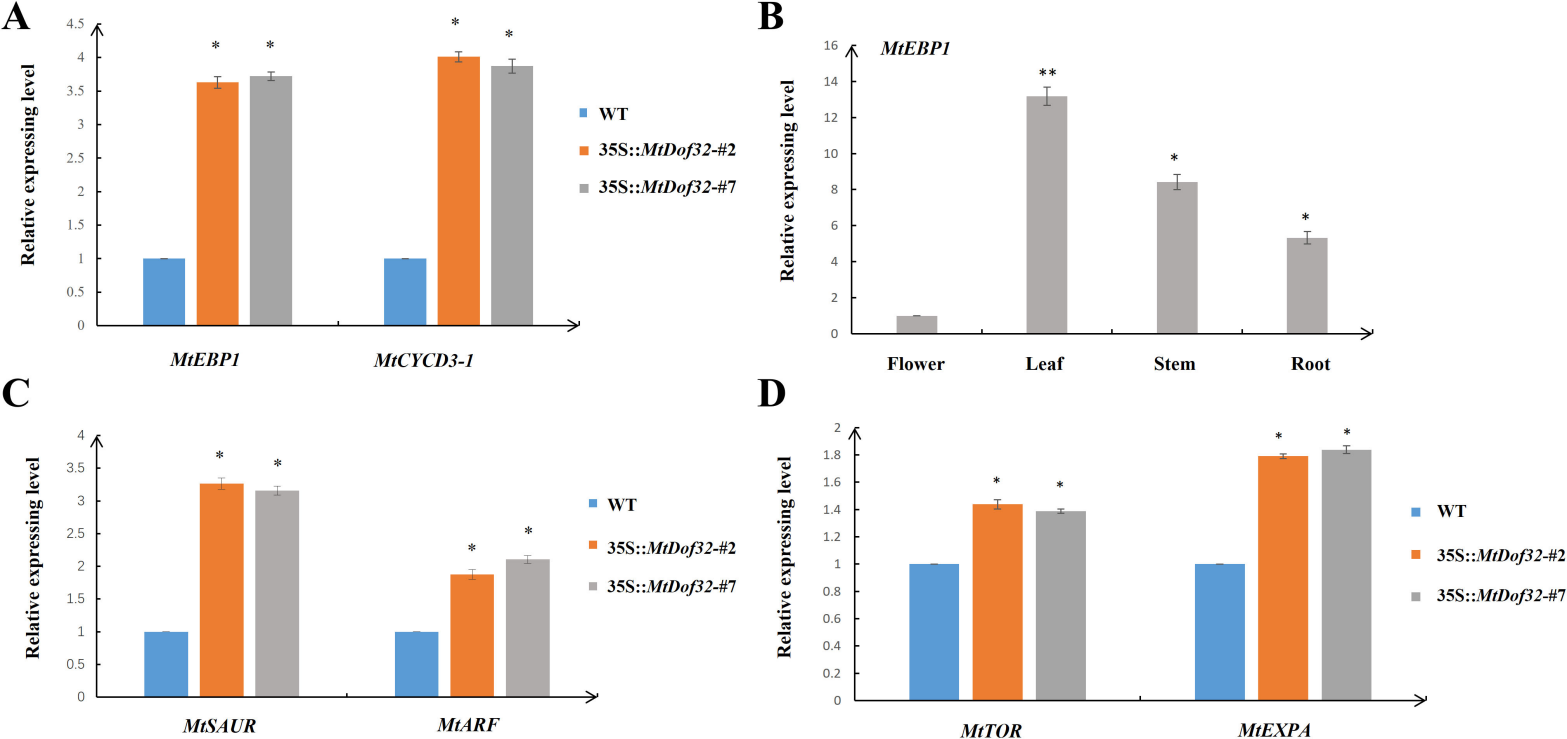
Molecular characterization of gene expression in *MtDof32*-overexpressing *Medicago truncatula*. **(A)** Expression levels of *MtEBP1* and *MtCYCD3–1* in wild-type (WT) and *MtDof32* transgenic plants. **(B)** Organ-specific expression profile of *MtEBP1* in wild-type *M. truncatula*. **(C)** Relative expression of auxin signaling-related genes *MtSAUR* and *MtARF* in leaves of wild-type and *MtDof32* transgenic lines. **(D)** Expression analysis of cell expansion-associated genes *MtTOR* and *MtEXPA* in leaves of wild-type and transgenic plants. *denotes statistically significant differences at p < 0.05. **denotes statistically significant differences at p < 0.01.

In *MtDof32* transgenic plants, both *MtEBP1* and *MtCYCD3–1* exhibited markedly elevated transcript levels compared to wild-type controls (**Figure 7A**). Furthermore, evaluation of the organ-specific expression profile in wild-type plants demonstrated that *MtEBP1* accumulates preferentially in leaf tissues (**Figure 7B**). Transgenic lines also showed substantial upregulation of key auxin signaling-related genes, including *MtSAUR* and *MtARF* (**Figure 7C**), along with increased expression of the cell expansion-related genes *MtTOR* and *MtEXPA* (**Figure 7D**). These consistent upregulation patterns across multiple functional categories suggest that *MtDof32* may collaborate with MtEBP1 to coordinate the regulation of genes involved in cell proliferation, auxin response, and cell expansion, providing mechanistic insights into its role in promoting organ growth.

## 4 Discussion

The Dof transcription factor family plays crucial regulatory roles in diverse plant-specific biological processes. Current research demonstrates their functional significance in multiple physiological pathways, including: photomorphogenesis and light signal transduction (Cai et al., 2024; Zheng et al., 2024), coordination of carbon-nitrogen metabolic networks (Gupta et al., 2015; Sun et al., 2025), embryogenesis and seed maturation (Da Silva et al., 2016), and abiotic/biotic stress responses (Jin et al., 2024; Wang et al., 2021). The model legume *M. truncatula* possesses 42 Dof transcription factors, yet the functional characterization of most members remains largely unexplored. In this study, we focused on *MtDof32* (Mtr7g010950), which encodes a 518-amino acid protein. Phylogenetic tree analysis revealed that MtDof32 shares the highest sequence similarity (72% identity) with L195_g008848 from *Trifolium pratense*. Consistent with its predicted function as a transcription factor, our previous subcellular localization studies using a pSAT6::*MtDof32*-GFP fusion construct demonstrated nuclear localization of the fluorescent signal (Guo et al., 2021).

qRT-PCR analysis revealed that *MtDof32* expression in *M. truncatula* responded dynamically to various exogenous hormone treatments, suggesting its potential involvement in hormone signal transduction cascades. Previous research results have shown that Dof could mediate responses to multiple hormones, including GA (Boccaccini et al., 2014), ABA (Zhai et al., 2022), SA (Kang et al., 2010), and MeJA (Hao et al., 2015). In buckwheat, the *FtDof* gene was downregulated under GA treatment. In *Helianthus annuus*, ABA treatment induced differential expression patterns among HaDof transcription factors, with distinct members showing either significant upregulation (e.g., *HaDof12*, *HaDof23*) or downregulation (e.g., *HaDof05*, *HaDof17*), suggesting functional diversification within this TF family during ABA signaling. This indicated that ABA had specificity in regulating *DOF* genes. In *Arabidopsis*, the expression of the *OBP2* was significantly increased after MeJA treatment. Plant hormone response profiling revealed that *FtDof* in *Fagopyrum tataricum* was specifically induced by MeJA treatment (3.5-fold increase), while showing suppression under ABA (0.4-fold), GA (0.3-fold), IAA (0.5-fold), and SA (0.6-fold) treatments compared to untreated controls (Li et al., 2022). Gibberellins typically inhibit the expression of *Dof*, while ABA and MeJA induce their expression more. This regulatory pattern may be related to the adaptive responses of plants under different physiological and environmental conditions.

Previous research has demonstrated that Dof transcription factors could function as regulators of flowering in plants. Some Dof members can directly or indirectly affect the expression of key flowering regulators such as *FT* and *CO* (Zhang et al., 2019). In this study, *MtDof32* transgenic *M. truncatula* displayed a pronounced late-flowering phenotype relative to wild-type plants. Further analysis revealed that *MtDof32* overexpression downregulated *MtFT* and *MtSOC1* expression, indicating its involvement in repressing flowering-related gene networks. Similar results have been found in our previously functional study of *MtDof32* in *Arabidopsis* (Guo et al., 2021). Studies in *Arabidopsis* demonstrated that AtCDF1 controlled flowering time through specific binding to the promoter regions of *CO* and *FT*, ultimately regulating their transcriptional activity (Zhang et al., 2019). Meanwhile, previous studies have also shown that auxin affected the initiation of flower bud differentiation by regulating gene expression. It can work synergistically with other hormones such as GAs and cytokinins to promote flower bud formation (Chandler and Werr, 2015). Auxin regulates flowering time by affecting the expression of flowering integrator genes such as *FT* and *SOC1*. High concentrations of auxin may delay flowering, while low concentrations may promote it (Yamaguchi et al., 2014). These data indicated that the function of MtDof32 was similar to that of some Dof family members, playing an important role in regulating plant flowering, and this process might involve different pathways.

The overexpression of *MtDof32* in *M. truncatula* led to a significant reduction in branch number, corroborating our earlier findings in *Arabidopsis thaliana* (Guo et al., 2021). Notably, transcript levels of *CAROTENOID CLEAVAGE DIOXYGENASE 7* (*CCD7*) and *BRANCHED1* (*BRC1*) were markedly elevated in *MtDof32*-overexpressing plants. *CCD7* encodes a carotenoid cleavage dioxygenase that catalyzes a key step in strigolactone (SL) biosynthesis, a phytohormone that suppresses lateral branching. BRC1, a TCP-family transcription factor, acts downstream of SL signaling to directly repress axillary bud outgrowth. Auxin affects the synthesis of SL by regulating the expression of *CCD7*, then indirectly regulate the expression of *BRC1*, and finally inhibit plant branching. Studies have shown that exogenous NAA or IAA could significantly upregulate the expression of *CCD7*, promote the synthesis of SL, and thus inhibit branching (del Rosario Cárdenas-Aquino et al., 2022; Chabikwa et al., 2019). Auxin also inhibits axillary bud outgrowth through transcriptional regulation of *BRC1* (Yan et al., 2020). These results collectively suggested that MtDof32 might regulate shoot branching through the auxin and SL pathway by modulating *CCD7* and *BRC1* expression.

The findings of this study suggested that overexpression of *MtDof32* in *M. truncatula* promoted cell expansion and regulated organ size, resulting in larger flowers and leaves. These results aligned with our previous observations in *Arabidopsis* (Guo et al., 2021). Additionally, yeast two-hybrid screening identified MtEBP1 (ERBB-3 BINDING PROTEIN 1) as a potential interacting partner of MtDof32 in *M. truncatula*. The physical interaction between MtDof32 and MtEBP1 was subsequently confirmed through yeast two-hybrid analysis. EBP1, a member of the peptidase M24 family, functions as an RNA-binding protein that regulates cellular growth and differentiation processes (Squatrito et al., 2006).

**Figure 8:**
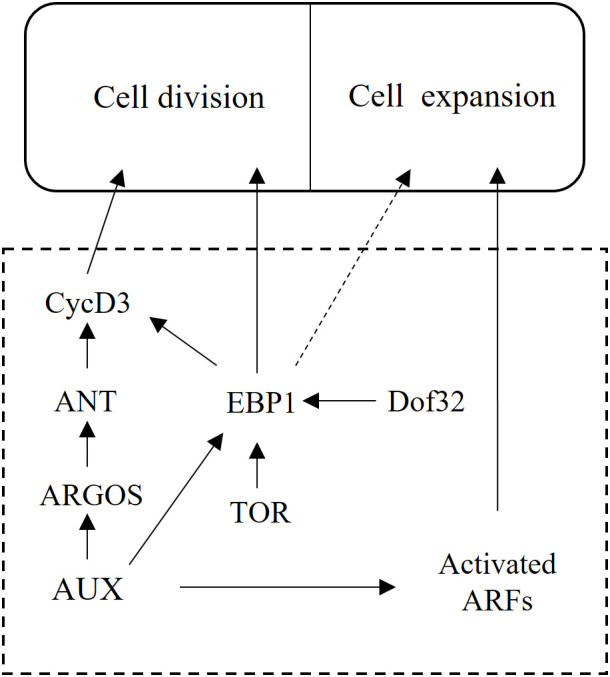
Proposed role of MtDof32-MtEBP1 in cell size regulation.

Previous studies have demonstrated that *Arabidopsis* EBP1 was involved in ribosome assembly and the inhibition of eIF2 phosphorylation, thereby influencing cell proliferation and growth (Eguchi et al., 2006; Squatrito et al., 2006). Research on *Solanum tuberosum* has shown that EBP1 promoted both cell proliferation and expansion during early organogenesis (Horváth et al., 2006). Heterologous expression of *Hevea brasiliensis HbEBP1* in *Arabidopsis* resulted in enlarged organs through prolonged vegetative growth (Cheng et al., 2016). Importantly, auxin has been found to regulate *EBP1* through cascade signaling, thereby controlling organ size (Magyar et al., 2012; Perrot-Rechenmann, 2010). Based on these findings, we hypothesize that MtDof32-MtEBP1 module regulates plant organ size through modulation of auxin signaling cascades (**Figure 8**). However, the precise regulatory mechanism by which this module influences cell size requires further investigation. Elucidating the function of this complex can significantly advance our knowledge to the molecular mechanisms of plant organ size regulation.

Furthermore, *MtSAUR* (Small Auxin Up RNA) and *MtARF* (Auxin Response Factor) are key components in the auxin signaling pathway. *MtARF* acts as a transcription factor that directly activates a suite of auxin-responsive genes, while *MtSAUR* proteins inhibit PP2C.D phosphatase activity, thereby activating plasma membrane H^+^-ATPases to induce cell wall acidification and loosening, which promotes cell expansion (Bao et al., 2024; Cancé et al., 2022). Their significant upregulation suggests that *MtDof32* may enhance the output of auxin signaling, thereby driving cell wall loosening and cell enlargement. Expansion A (EXPA) is a class of non-enzymatic cell wall-loosening proteins that disrupt hydrogen bonds between cellulose microfibrils and matrix polysaccharides (such as xyloglucan), inducing cell wall “creep” and irreversible extension (Sun et al., 2021). The observed upregulation of *MtEXPA* in transgenic plants is consistent with previously reported mechanisms by which EXPAs promote cell expansion (Chen et al., 2021), indicating that *MtEXPA* likely acts as a direct executor of cell wall loosening, leading to increased cell size and organ enlargement. TOR (Target of Rapamycin) has been shown to regulate *EBP1* expression levels, and overexpression of both *EBP1* and *TOR* can impact plant organ size (Jamsheer et al., 2022). Taken together, these results suggest that *MtDof32*, as a transcriptional regulator, may directly or indirectly activate the expression of *MtSAUR*, *MtARF*, *MtEXPA* and *MtTOR*, thereby integrating hormonal signals with cell wall remodeling at the transcriptional level to promote cell expansion and organ growth.

Organ morphogenesis in plants requires the tight spatiotemporal coordination of cellular proliferation and expansion (Gonzalez et al., 2012). Existing research indicated that plant hormones played a pivotal role in regulating organ size. These hormones formed a complex signaling network that translated external or internal stimuli into developmental responses (Zhu et al., 2020). Among the diverse types of plant hormones, auxin, cytokinin, brassinosteroids, and gibberellins were particularly significant, each with distinct yet interconnected roles in modulating plant growth and development (Santner and Estelle, 2009). Auxin, in particular, was crucial for plant development, exerting multifaceted effects on organ size. Auxin regulated flowering time and branching patterns in plants by controlling the expression of flowering-related genes (such as the *FT* gene) and apical dominance (through polar transport and PIN protein-mediated signaling), respectively, while interacting with other hormones (such as GA, ethylene, and cytokinins) to coordinately regulate plant growth and development (Chandler and Werr, 2015; Cucinotta et al., 2021; Hu et al., 2022; Dubois et al., 2018). In this study, the altered flowering time (including key genes in the flowering pathway) and branching number observed in overexpressing *MtDof32* plants might be attributed to the modulation of auxin signaling by the MtDof32-MtEBP1 module. This suggested that the MtDof32-MtEBP1 interaction could influence auxin-mediated signaling pathways, thereby impacting plant development.

The observed phenotype, characterized by significantly enlarged leaves yet reduced branch number and overall biomass in *MtDof32*-overexpressing plants, can be interpreted through the lens of source-sink balance and resource allocation. While *MtDof32* promotes cell expansion, leading to larger individual leaves—potentially enhancing photosynthetic capacity (source strength)—it concurrently strongly upregulates key branching suppressors such as *MtCCD7* and *MtBRC1*. This results in a severe reduction in sink number and capacity. We propose that the significantly diminished sink demand imposes a feedback inhibition on photosynthetic activity, ultimately constraining overall biomass accumulation despite the increase in leaf size. This illustrates a classic trade-off where the genetic enhancement of one organ comes at the cost of another, likely mediated through auxin signaling pathways involving MtEBP1, and potentially modulated by the TOR signaling network (Jamsheer et al., 2022).

From a practical perspective, *MtDof32* may not serve as a universal “yield-increasing” gene for biomass crops but rather as a valuable “plant architecture shaping” tool. Its application potential lies in precision breeding strategies, such as improving leaf yield in vegetable crops (e.g., lettuce, tobacco) or developing compact, high-density planting ideotypes with reduced branching. Future efforts could explore tissue-specific promoters to spatially control its expression, thereby uncoupling its beneficial effect on leaf growth from its inhibitory effect on branching.

## 5 Conclusion

Based on the findings presented, we proposed that MtDof32 and MtEBP1 functioned as a protein complex to regulate cell size in *M. truncatula*, a process that likely involve plant hormone signaling pathways. However, as upstream regulatory factors, the specific downstream genes targeted by MtDof32 and MtEBP1, as well as the mechanisms underlying their regulation, remain to be elucidated. Further experimental studies are necessary to explore the roles of MtDof32 and MtEBP1 within the molecular networks that govern cell size and to uncover the precise regulatory patterns involved.

## Data Availability

The original contributions presented in the study are included in the article/**Supplementary Material**. Further inquiries can be directed to the corresponding author.
