# Supplementary material for: Overexpression of MtDof32 in Medicago truncatula enhances leaf and flower organ size through modulation of cell expansion
Source: Front Plant Sci. 2025 Dec 4;16:1666846. doi: 10.3389/fpls.2025.1666846 (PMC12711816; doi:10.3389/fpls.2025.1666846)
Supplement: Supplementary file 1 [file DataSheet1.docx]

Supplementary Material

**Supplementary Table 1.** Primer sequences used for gene cloning, plasmid construction and qRT-PCR.

| **Primer name** | **Primer sequence(5′-3′)** | | |
| --- | --- | --- | --- |
|  | **F** | **R** |  |
| **For gene clone** |  |  | |
| MtDof32 | ATGACAAACCGCAAAGACAAAGT | ATGAAAGAAAGAAGCCAGGAAAG | |
| MtEBP1 | ATGTCGGATGATGAAAGGGA | TTCCTGAGATGTGGCCTCAT | |
| **For GFP fusion** |  |  | |
| SAT- EBP1 | CACCATTTACGAACGATAGCATGTCGGATGATGAAAGGGA | CTAGTCAGATCTACCATCCCTTCCTGAGATGTGGCCTCAT | |
| **For overexpression** |  |  | |
| 3302-Dof32 | GAACACGGGGGACTCTTGACATGTTGGAAACAAAAGACTCT | TTTACCCTCAGATCTACCATCGAAGTCTCGTGAAAGTGAAGC | |
| **For identification** |  |  | |
| p3302 | TGACGCACAATCCCACTATCCTT | CGTATTAAATGTATAATTGCGGGAC | |
| **For qRT-PCR** |  |  | |
| qMtDof32 | AATAGTTCCTTGTCCTCGCTG | CCGGTACGTTTCTCATGGTTC | |
| qMtFTa1 | TATGAAAGGCCACGACCCAC | GGTTCTTCCTCCAGAGCCAC | |
| qMtSOC1 | TGAACGATACCGCAGTCATACCA | CAAGACCCTAAACCTTCTCCTAA | |
| qMtEBP1 | CTGCTAGGGCTTTGGAGGAG | GGCTAGCCATGCCTTGATCT | |
| qMtCCD7 | ACCCCGTAGTTGCTTCATGG | GGTATCCATGGCCGTCTAGC | |
| qMtBRC1 | GGGAAGCATACAACAACCGC | TCTGAGCCTGACTGACACAA | |
| qMtSAUR | CTCATTACATCCTATACTCTAGGCACT | CACTTGGTATGGAGCAACTTTCA | |
| qMtARF | GTTGCGAAAGATCAAACAAATCC | GAAGATAGTTCCTTGTAAGTGCC | |
| qMtEXPA | GAATCGTCCCTGTAGCCTTTCGC | CATTGGCATCCACCCTGTTTGAG | |
| qMtTOR | GAGGAAGATTCGTGGATTAGGAT | CAAACAGCCGTATGTGAGGATTA | |
| qMtCYCD3-1 | TGTGTTGGCCACTGCTACAA | ATGATGCTCCCACTGTCCAC | |
| qMtActin | TGGGCTGCCACAGAACATTTGA | GCTGTGGTTGCTTTTTTGGTGTCTC | |
| **For yeast hybridization** |  |  | |
| pGBKT7-MtDof32 | AGGAGGACCTGCATATGGCCATGTTGGAAACAAAAGACTCT | ATCCCCGGGAATTCGGCCTCTTACGAAGTCTCGTGAAAGTGAAGC | |
| pGADT7-MtEBP1 | GAGGCCAGTGAATTCCACCCATGTCGGATGATGAAAGGGA | TCCCGTATCGATGCCCACCCTCATTCCTGAGATGTGGCCT | |
| **For BIFC** |  |  | |
| Dof32-YFPn | GACCTCGAGGGTACCGCTCCCATGTTGGAAACAAAAGACTCT | ATCAACTTTTGCTCCATCCCCGAAGTCTCGTGAAAGTGAA | |
| EBP1-YFPc | ACCTCGAGGGTACCGCTCCCATGTCGGATGATGAAAGGGA | ACATCGTATGGGTACATCCCTTCCTGAGATGTGGCCTCAT | |

**Supplementary Table 2.** Screening of *Medicago truncatula* cDNA yeast library by MtDof32.

| **Bait protein** | **Interaction protein gene ID** | **Interaction protein gene name** | **Number** |
| --- | --- | --- | --- |
| MtDof32 | MTR_2g099360 | Peroxisomal membrane protein 13 | 3 |
| MtDof32 | MTR_7g069390 | Erbb-3 binding protein 1 | 10 |
| MtDof32 | MTR_3g110340 | Protein cereblon | 1 |
| MtDof32 | MTR_4g074350 | Delta(24)-sterol reductase | 1 |
| MtDof32 | MTR_5g084570 | Abscisic acid and environmental stress-inducible protein | 2 |
| MtDof32 | MTR_7g088740 | 4-alpha-glucanotransferase DPE2 | 1 |
| MtDof32 | MTR_7g095190 | tRNA (guanine-N(7)-)-methyltransferase non-catalytic subunit wdr4 | 1 |
| MtDof32 | MTR_1g022295 | Maf-like protein DDB | 6 |
| MtDof32 | MTR_7g088410 | Alpha-L-arabinofuranosidase 1 | 2 |
| MtDof32 | MTR_8g021310 | Transmembrane protein 87A | 1 |
| MtDof32 | MTR_8g078870 | Oxygen-evolving enhancer protein 1 | 2 |


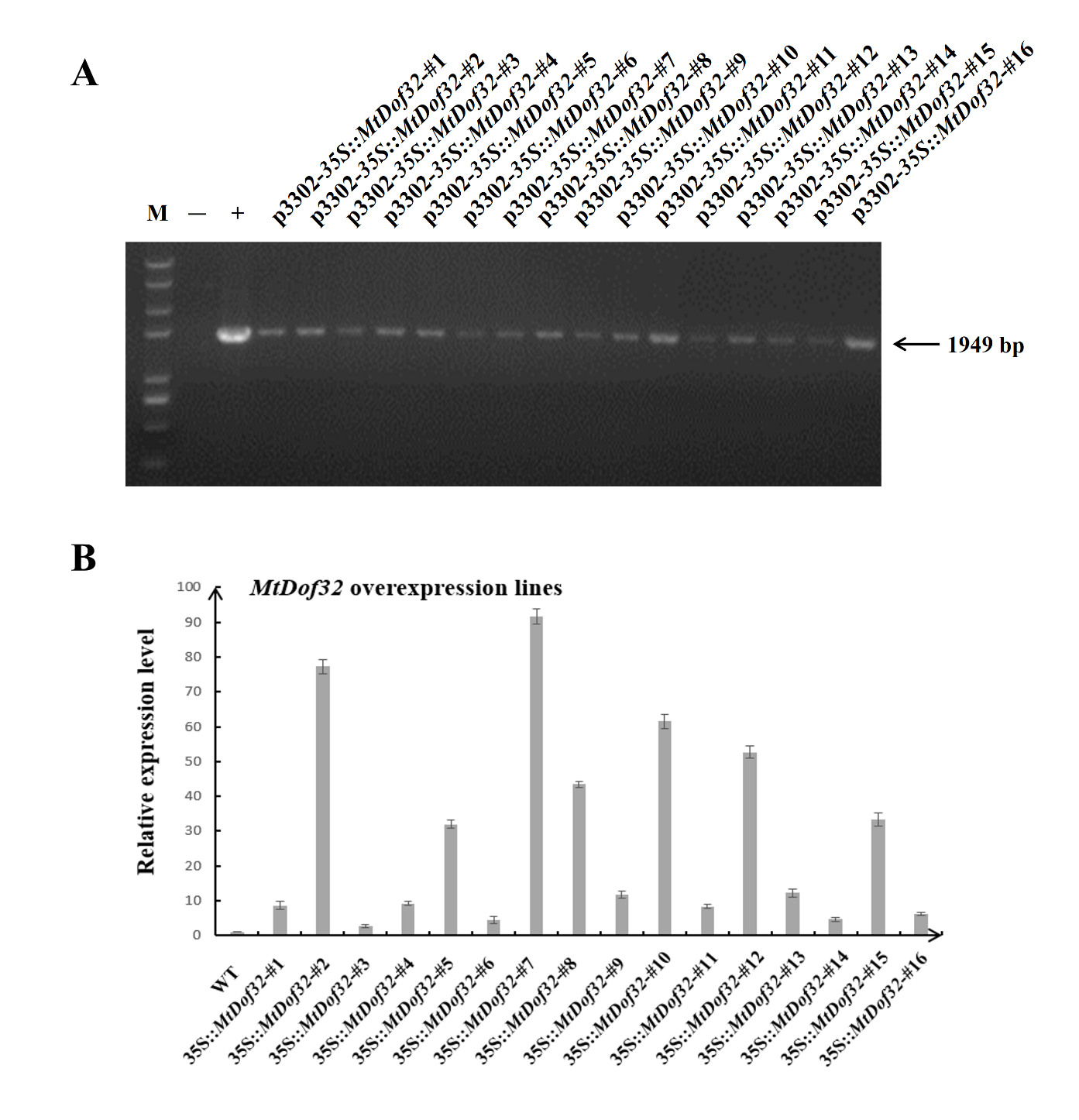


**SUPPLEMENTARY FIGURE 1.** Identification of positive seedlings and qRT-PCR detection in *MtDof32* overexpression *Medicago truncatula*

(A) Identification of *MtDof32* overexpressing *Medicago truncatula* positive plants. M means Marker, “-” means negative control, “+” means positive control, p3302-35S::*MtDof32*#1~16 were different transgenic *Medicago truncatula* plants. (B) The relative expression level of *MtDof32* in transgenic *Medicago truncatula* was measured by qRT-PCR. *MtActin* (Mtr3g095530) was used as an internal reference gene. The final data was relative to the expression level of *MtDof32* in wild type plants*.* All experiments were replicated three times. Data are shown as the mean values ± SD.


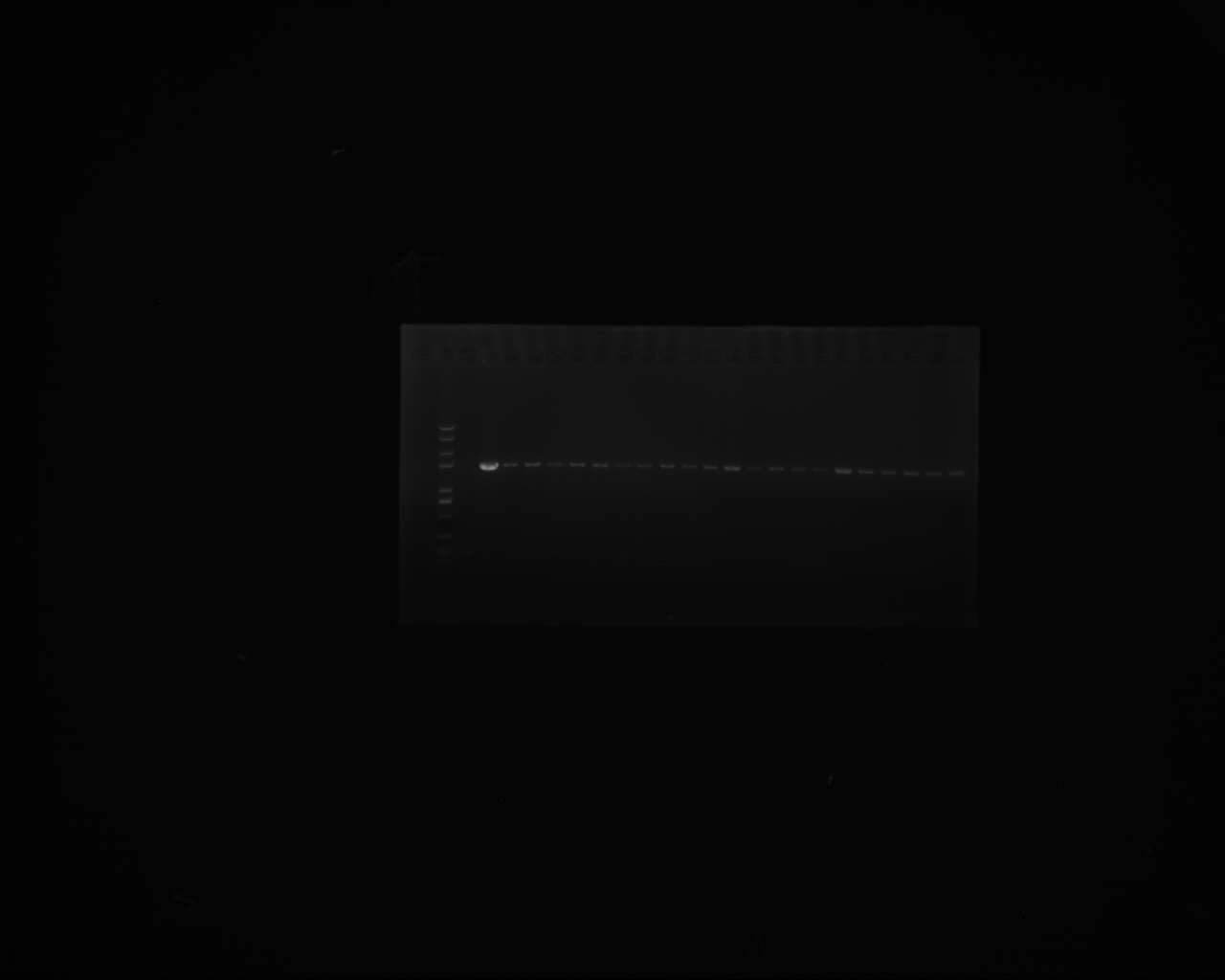


**SUPPLEMENTARY FIGURE 2.** A full scan of the entire original gel of Supplementary Figure 1 (A).

During the agarose gel electrophoresis analysis, the original gel image exhibited insufficient band visibility. Therefore, we have adjusted the brightness of the image in Supplementary Figure 1(A) to enhance clarity while ensuring that all modifications strictly maintain the original data integrity. The last five samples in the image correspond to positive *MtDof32* transgenic *Arabidopsis* plants and were therefore excluded from Supplementary Figure 1(A).


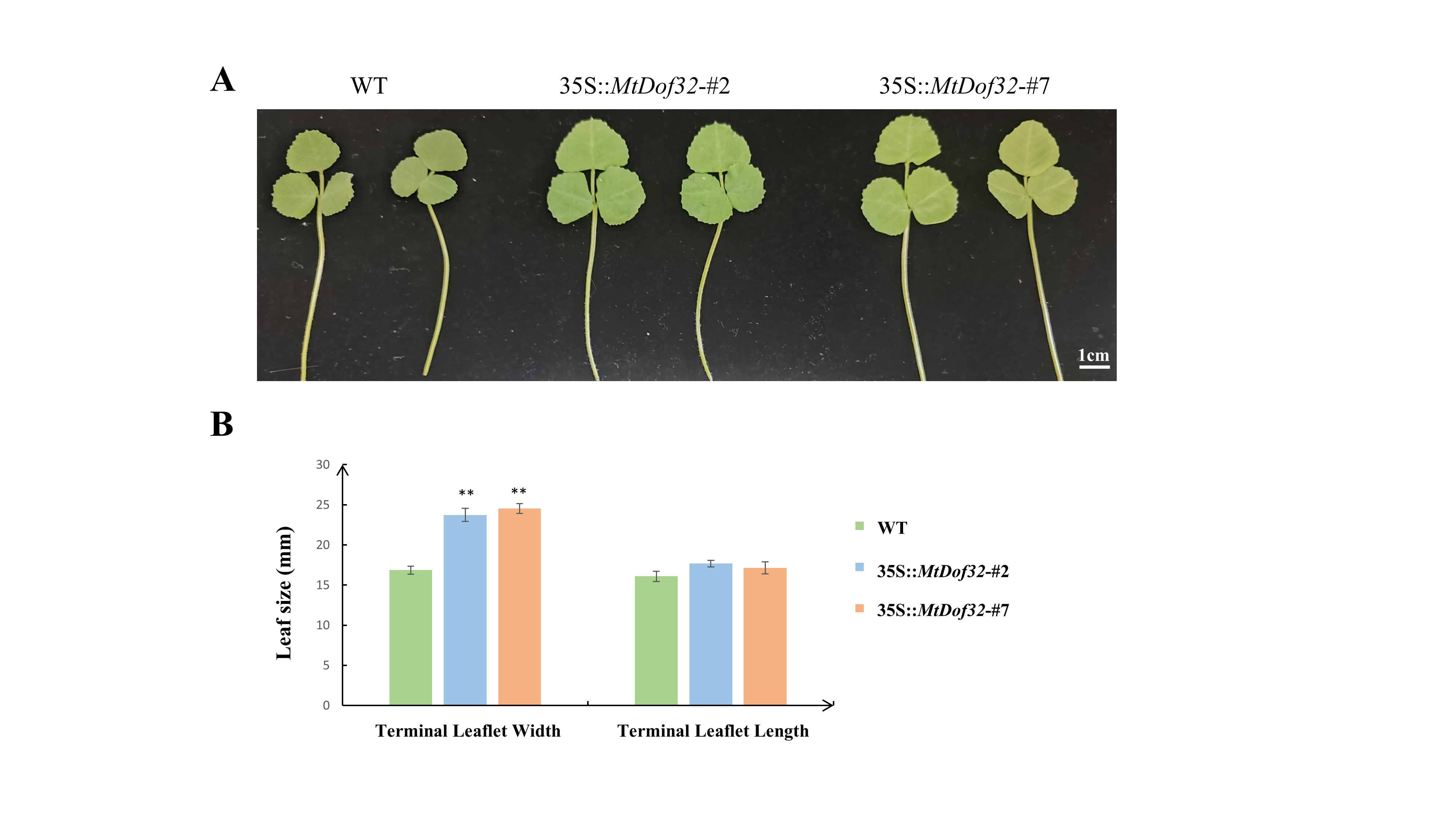


**SUPPLEMENTARY FIGURE 3.** Comparative analysis of leaf between *MtDof32* transgenic plants and wild *M. truncatula* at 30 days of growth.

(A) Comparative analysis of leaf between *MtDof32* transgenic plants and wild *M. truncatula*. Samples were collected at 30 days of growth. (B) Comparison of terminal leaf size between wild type and *MtDof32* transgenic group *M. truncatula* (n=18). *, denotes statistically significant differences at p < 0.05.


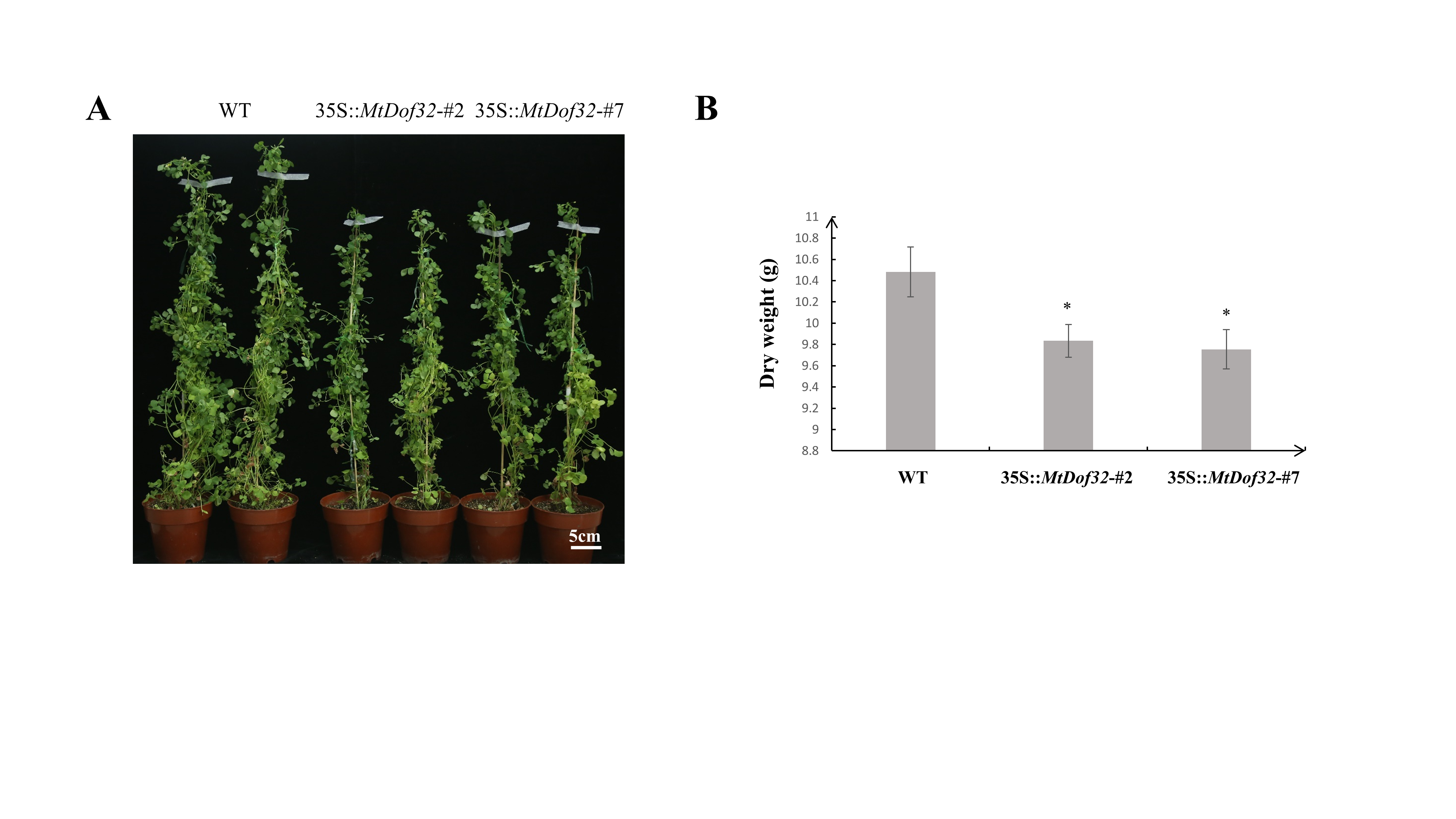


**SUPPLEMENTARY FIGURE 4.** Comparative analysis of biomass between *MtDof32* transgenic plants and wild *M. truncatula* at 90 days of growth.


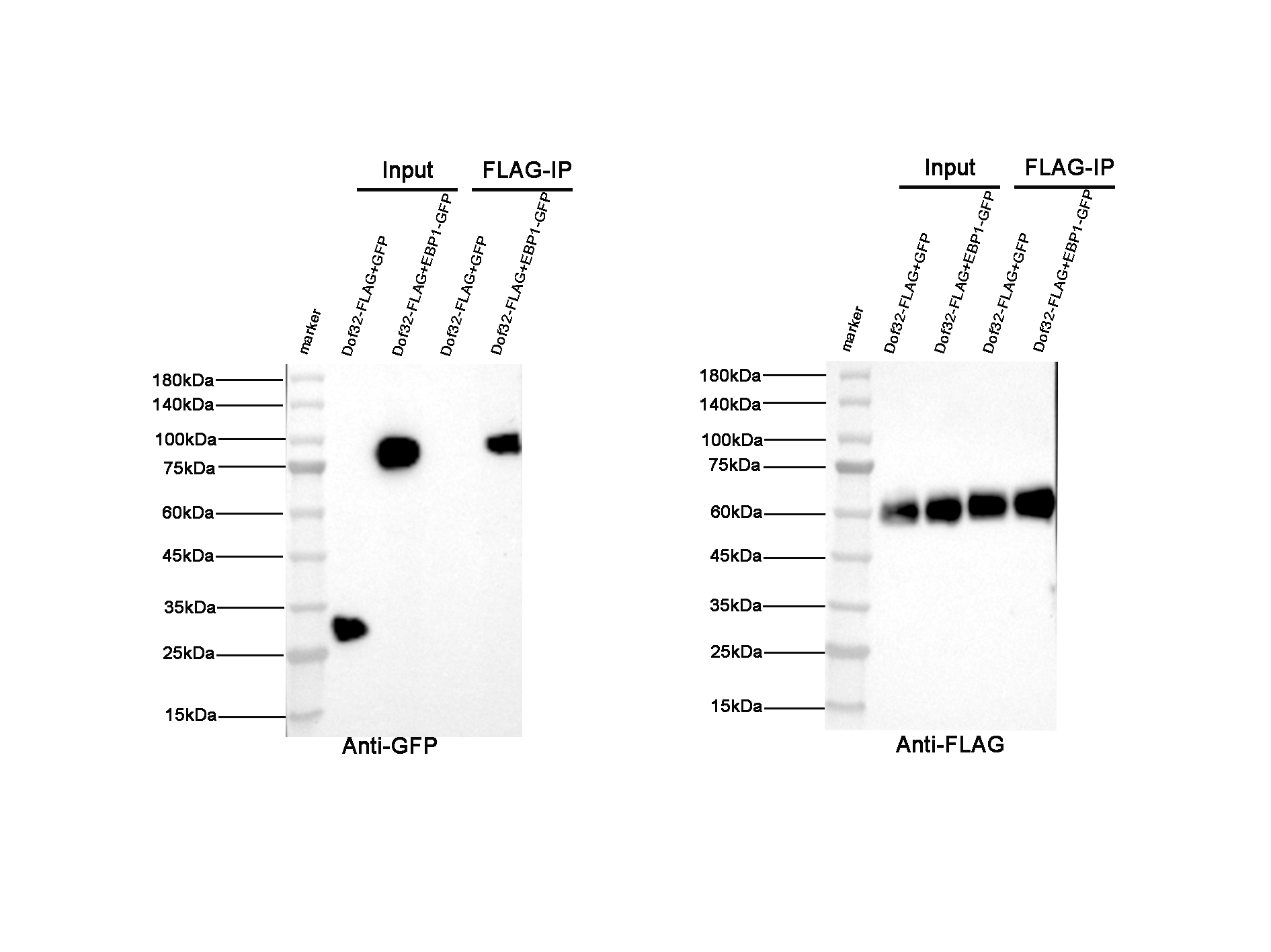


**SUPPLEMENTARY FIGURE 5**. Dof32 can interact with EBP1 in Co-IP experiments. The constructs 35S:*Dof32*-FLAG and 35S:*EBP1*-GFP were co-expressed in tobacco leaves. Input samples, representing total protein extracts, were analyzed by Western blotting to assess the expression levels of FLAG-Dof32 and GFP-EBP1. The products of FLAG-IP were probed with anti-FLAG and anti-GFP antibodies to confirm the interaction.
